# A review of adjuvants for *Leishmania* vaccine candidates

**DOI:** 10.1016/S1674-8301(10)60004-8

**Published:** 2010-01

**Authors:** Joshua M. Mutiso, John C. Macharia, Michael M. Gicheru

**Affiliations:** a Department of Tropical and Infectious Diseases, Institute of Primate Research, National Museums of Kenya, P.O. Box 24481-00502, Karen,Nairobi, Kenya; b Department of Zoological Sciences, School of Pure and Applied Sciences, Kenyatta University, P.O. Box 43844-00100, Nairobi, Kenya.

**Keywords:** adjuvants; *Leishmania* vaccines; immune responses; leishmaniasis.

## Abstract

Over the last decade, there has been a flurry of research on adjuvants for vaccines, and several novel adjuvants are now licensed products or in late stage clinical development. The success of adjuvants in enhancing the immune response to antigens has led many researchers to re-focus their vaccine development programs. Although several vaccine candidates have been tested against leishmaniasis, there is yet no effective vaccine against this parasitic disease. Recent research has documented that efforts to develop effective *Leishmania* vaccine have been limited due to lack of an appropriate adjuvant. In view of this, this review paper outlines some of the adjuvants that have been used in *Leishmania* vaccine candidates and cites a few of the responses obtained from these studies. The aim of the present review is to consolidate these findings to facilitate the application of these adjuvants in general and experimental vaccinology.

## INTRODUCTION

Leishmaniases are parasitic diseases caused by protozoan flagellates of the genus *Leishmania*. The parasite infects numerous mammal species, including humans, and is transmitted through the infective bite of an insect vector, the sandfly of the genus *Phlebotomus* in the Old World or *Lutzomyia* in the New World[1].With the disease having 14 million clinical cases on a worldwide scale and 350 million people at risk of infection[2], urgent control measures are needed to stop the over 2 million infections reported each year. *Leishmania* parasites cause three main forms of leishmaniases according to the localization of the parasites in mammalian tissues, notably visceral, cutaneous, and mucocutaneous leishmaniasis[3]. Cases of self cure in cutaneous leishmaniasis (CL), accompanied by immunity to reinfection, make vaccine development a feasible leishmaniasis control method. Over the years, some of the antigens tested include live vaccines/leishmanization, killed vaccines, live-attenuated vaccines, recombinant and synthetic vaccines, naked DNA vaccines and vector-derived vaccines. However, so far, there is no vaccine against leishmaniasis in routine use anywhere in the world[4].

In all forms of leishmaniasis, a cure is affected through a cellular immune response involving interferon gamma (IFN-γ) that activates host macrophages to eliminate the parasite[5]. So far, no *Leishmania* vaccine candidate has been able to induce the required potent, effective and safe cell mediated immune response that can confer effective immunity against the parasite. The choice of antigen and adjuvant are of paramount importance when conducting a vaccine trial for end-point use in humans[6]. Successful vaccine development requires knowing which adjuvants to use and knowing how to formulate adjuvants and antigens to achieve stable, safe and immunologic vaccines[7]. Earlier investigators have used the Th 1 and Th 2 paradigm as a strategy for the selection of an antigen in vaccine development against leishmaniasis[5],[8]. Thus, leishmanial antigens that predominantly stimulate Th 1 responses in patient cells or mice infected with the parasite have been accepted as “potential protective antigens” and therefore promising vaccine candidates[5],[9]. Recombinant and killed *Leishmania* antigens are the safest vaccines but they require the addition of an appropriate adjuvant for them to induce a protective Th 1 immune response[10]. Some studies indicate that, efforts to develop effective *Leishmania* vaccine have been limited due to lack of such an appropriate adjuvant[11].

The term adjuvant has been used for any material that can increase the humoral or cellular immune response to an antigen. In the conventional vaccines, adjuvants are used to elicit an early, high and long-lasting immune response. The mode of action of adjuvants was described[12] as: the formation of a depot of antigen at the site of inoculation, with slow release; the presentation of antigen to immunocompetent cells; and the production of various and different lymphokines. Although Mayrink *et al*[13] used the intramuscular route in man with killed parasites without an adjuvant, and successful protection without adjuvants has been achieved in a number of animal studies, it is probable that vaccines utilizing subcellular components or purified macromolecules may require the use of suitable adjuvants. Studies carried out in animal models for leishmaniasis have demonstrated the requirements for an adjuvant in *Leishmania* vaccines.

This review article is based on previous experimental studies and it attempts to outline some of the adjuvants that have been tested in Leishmania vaccines and describes briefly some of the immune responses induced by these antigen-adjuvant vaccines.

## ADJUVANTS THAT HAVE BEEN TESTED IN *LEISHMANIA* CANDIDATE VACCINES INCLUDE THE FOLLOWING:

### Interleukin-12 (IL-12)

The adjuvant potential of IL-12 for a vaccine against leishmaniasis has been documented in the murine model. Mice immunized with soluble *Leishmania* antigen (SLA)+IL-12 were completely protected against disease. IL-12 in the presence of SLA induces differentiation of CD4^+^Th 1 cells in the lymph node and spleen. Mice immunized with SLA+IL-12 produced little IL-4 but large amounts of IFN-γ that were comparable to those observed in resistant C3H/HeN mice. Lesions from unimmunized BALB/c mice or mice that had been immunized with SLA alone contained greater than 10^7^ parasites, whereas BALB/c mice immunized with SLA+IL-12 contained 10^3^ parasites[14]. In studies using the Vervet monkey, low doses of a human recombinant IL-12 preparation induced a small increase in the parameters of cell-mediated immunity, similar to animals that received antigen without IL-12[15].

IL-12 produced by macrophages, dendritic cells, B-lymphocytes and other accessory cells is a critical component in the development of cell-mediated immunity and stimulates proliferation and production of IFN-γ from T-cell and natural killer cells[16],[17], which is important for protective immunity in leishmaniasis[18]. Furthermore, this cytokine has the ability to direct the development of CD4+Th 1 cells which are necessary for protective immunity in leishmaniasis[14],[17],[19],[20]. It has been demonstrated in experimental animal models that a dominant Th 1 lymphocyte response (IL-2, IFN-γ) is associated with self-limiting disease, whereas a dominant Th2 response (IL-4, IL-5) is linked to progressive disease[21]. Addition of IL-12 to leishmanial antigens [thiol-specific antioxidant (TSA), Leishmania elongation and initiation factor (LeIF) and Leishmania major (L. major)stress-inducible protein (LmSTI-1)] resulted in complete protection of susceptible mice against progressive disease, whereas no protection was observed in the absence of adjuvant. A limiting factor for use of IL-12 may be the high cost. IL-12 is expensive and difficult to manufacture and its efficacy and safety as an adjuvant for human use is questionable[22].

### Granolucyte macrophage-colony stimulating factor (GM-CSF)

Recombinant GM-CSF has been documented to have a potent cytokine adjuvant effect based on its activity of inducing activation, maturation and migration of dendritic cells[23]. This cytokine is known to yield a dominant Th 1 type response[24]. It has been shown to be effective in doses of 25 mg to 50 mg as an adjuvant[25], and it has been shown to be well tolerated when used in patients with visceral leishmaniasis[26]. A cocktail vaccine for immunotherapy of mucosal leishmaniasis patient containing 5 mg of each of the antigens, TSA, LmSTIl, rLbhsp83 and 10 mg of LeIF in combination with 50 mg of GM-CSF (Leukine^®^) added as an adjuvant showed no adverse reaction, except in one healthy subject who had erythema and induration at the injection site that was considered to be a secondary infection, but possibly due to an intense delayed type hypersensitivity (DTH) response. The cytokine was found to promote wound healing in vaccinated patients suffering from mucosal leishmaniasis[24]. When used at high doses and injected at the site of the lesion and in combination with pentavalent antimony, GM-CSF shortens the healing time[27].

### Bacille Calmette Guérin (BCG)

BCG is a weakened (attenuated) version of the bacterium *Mycobacterium bovis*, which is closely related to *Mycobacterium tuberculosis*, the agent responsible for tuberculosis (http://www.MedicineNet.com). BCG has been clearly associated with the induction of a Th 1 immune response[28], and is probably the most acceptable Th 1-inducing adjuvant presently available for use in humans. BCG has been used successfully for anti-*Leishmania* immunotherapy in South American patients without side effects. BCG vectors carrying gp63 have also been used successfully to induce protection in *L. major*-infected murine models[29],[30]. In some of the early studies on *Leishmania* killed vaccines, a combination of killed *L. mexicana* or *L. braziliensis* promastigotes and *Mycobacterium bovis* BCG was used by convict and colleagues both prophylactically and therapeutically against South American leishmaniasis[31]. When used in the therapeutic mode, vaccination appeared to induce a high cure rate even in patients with severe cases. Cure was accompanied by the development of Th1-type immune responses in the recipients, with the production of IFN-γ and the absence of IL-4[31],[32].

Vaccination with BCG plus killed *Leishmania* promastigotes reduced acute infection by *T. cruzi* in mice, increasing survival time and decreasing parasitaemia and mortality[33]. Sohrabi *et al*[11] found that liposomes containing autoclaved *L. major* (ALM) antigens mixed with BCG could be used to induce a Th1 response in resistant C57 BL/6 mice. Complete protection of *Leishmania donovani (L. donovani)* infected Indian langurs was obtained by vaccination with alum-precipitated ALM combined with BCG at a dose of 1 mg per animal[34]. A study by Momeni *et al*[35] in Iranian children with zoonotic cutaneous leishmaniasis concluded that a single dose vaccination with killed *L. major* vaccine plus BCG, though safe, was not sufficiently immunogenic to provide a measurable response when compared to the BCG alone. Hence multiple doses or other adjuvants were suggested as ways of increasing immunogenicity to this antigen. The application of BCG as an adjuvant in vaccines has not always been without problems. Adverse effects or complications have been reported with the use of BCG, including inflammatory arthritis and autoimmune reactions[34],[36]–[38]. Thus, these effects of BCG make it quite undesirable for a safe vaccine.

### Montanide ISA 720

Montanide Incomplete Seppic adjuvants, (Montanide ISA) are a group of oil/surfactant based adjuvants in which different surfactants are combined with either a non-metabolizable mineral oil, a metabolizable oil, or a mixture of the two oils (http://www.nal.usda.gov/awic/pubs/antibody/overview.htm). Montanide ISA 720 has been approved for experimental use in humans as an alternative adjuvant to aluminium hydroxide[6],[39],[40]. It has been shown to be immunogenic, inducing both Th 1-type cellular and humoral immune responses in humans[6]. Montanide ISA 720 has also shown good results in non-human primate vaccination studies[6],[41]. A safety and immunogenicity study of a malaria vaccine containing single, intramuscular doses of ICC-1132 formulated in Montanide ISA 720 (ICC-1132/ISA 720) showed that the vaccine was safe and well tolerated. Transient injection site pain was the most frequent complaint. All vaccines that received either 20 µg or 50 µg of ICC-1132/ISA 720 developed anti-immunogen antibodies, predominantly of opsonizing immunoglobulin G (IgG) subtypes. Peripheral blood mononuclear cells (PBMC) of ICC-1132/ISA 720 vaccinees proliferated and released cytokines (IL-2 and IFN-γ) when stimulated with recombinant Plasmodium falciparum CS protein, and CS-specific CD4^+^ T-cell lines were established from volunteers with high levels of antibodies to the repeat region[40].

Montanide ISA 720 adjuvant was also used in an HIV vaccine performed to examine the safety and immunogenicity of a multi-epitope polypeptide comprising the central 15 amino acids of the V loop from six HIV-1 isolates. This protein, called TAB9, was emulsified in Montanide ISA 720 (Seppic, Paris) and administered intramuscularly at doses of 0, 0.2 and 1 mg to 24 healthy, HIV-1 seronegative adult males. Three immunizations were given at months 0, 1 and 6 in a randomized, double blind, placebo controlled clinical trial[39]. The placebo was generally well tolerated. However, severe local reactions were observed in TAB9 vaccinated subjects after the second and third inoculations. Seven out of eight volunteers from the lower dose group showed moderate or severe local inflammation, while four out of eight subjects from the higher dose group developed granulomas and sterile abscesses. The reactogenicity depended on the number of inoculations given and the dose of TAB9. Both doses were immunogenic, all immunized volunteers seroconverted and antibodies were broadly reactive against the V3 peptides included in the protein[39] . In *Leishmania* infections, the use of Montanide avoids problems that have been encountered with other adjuvants (for example, Freund's), including adverse reactions[42],[43]. Vervet monkeys immunized with a combination of recombinant glutathione-S-transferase-Histone-1 (*Leishmania* antigen) and Montanide ISA 720 adjuvant were able to generate a durable cellular response that was sufficient to control infection in the majority of the monkeys[6]. A vaccination study combining the use of *L. major* exogenous antigens and Montanide ISA 720 in mice did not show an increase in protection by the adjuvant[44].

### Aluminium salts

Aluminium salt based adjuvants, referred to generally as “Alum”, are non-crystalline gels based on aluminium oxyhydroxide, aluminium hydroxyphosphate or various proprietary salts such as aluminium hydroxyl-sulfate[7]. Among the adjuvants approved for use in humans, alum has had the greatest clinical use and is relatively non-reactogenic[45]. Aluminium phosphate and aluminium hydroxide (alum) are the mineral compounds most commonly used as adjuvants in human vaccines[46]. The use of alum was applied more than 70 years ago by Glenny *et al*.[47], who discovered that a suspension of alum-precipitated diphtheria toxoid had a much higher immunogenicity than the fluid toxoid. Aluminium adjuvants have been thought to form a repository of antigen in tissue, to produce particulate antigen for presentation to immune cells, and perhaps to activate complement and other immune enhancers. The immune response to some, but not all, protein antigens is enhanced by aluminium salts; however, these salts have little effect on peptide and polysaccharide antigens. Aluminium adjuvants enhance the primary immunization series, reducing the amount of antigen needed per dose and the number of required doses[48].

In human vaccination, aluminium adjuvants have been primarily used in tetanus, diphtheria, pertussis and poliomyelitis vaccines and more recently also hepatitis A and hepatitis B virus vaccines. In addition, other aluminium-adsorbed vaccines are available for special risk groups. For example, an aluminium-adsorbed anthrax vaccine is administered to military servicemen in the USA[49]. A study in the mouse model indicated the immunity elicited by killed *Leishmania* antigen plus mouse rIL-12 without alum may be relatively short-lived, lasting less than three months[50]. A vaccine with autoclaved *Leishmania amazonensis (L. amazonensis)* promastigotes using rhIL-12 and alum as adjuvants was safe and fully effective in a Rhesus monkey model of CL[10]. Alum-precipitated, ALM vaccine combined with BCG was shown to induce successful vaccination against *L. donovani* infected Indian langurs[34]. In a recent study, cells harvested from mice immunized with *L. major* soluble exogenous antigens (SEAgs) plus mouse rIL-12 plus alum showed enhanced proliferative responses and more cytokines than cells from mice immunized with *L. major* SEAgs alone[44]. Adverse reactions that have been reported with aluminium-containing vaccines are generally local reactions, including sterile abscesses, erythema, subcutaneous nodules, granulomatous inflammation, and contact hypersensitivity[51].

### Monophosphoryl lipid A

The Bill and Melinda Gates Foundation has funded the development of a chimeric vaccine made of three recombinant leishmanial antigens (LeIF, LmSTI-1 and TSA) in the form of a fusion protein combined with monophosphoryl lipid A plus squalene (MPL^®^-SE) in squalene oil as adjuvant. Phase I trials of this vaccine in healthy volunteers in the USA and initial efficacy testing as a therapeutic vaccine in patients in Latin America suggest satisfactory vaccine safety and immunogenicity. The most effective combination of Leish-111f, which is formulated with 20 µg of MPL^®^-SE, affords protection for over 14 weeks.

The Leish-111f-MPL^®^-SE vaccine has been evaluated extensively in preclinical safety and toxicology studies in five animal species, with no adverse effects being observed[52].

In another recent study, the immune response and protection induced by Leish-111f formulated with MPL^®^-SE demonstrated that, mice developed strong humoral and T-cell responses to the vaccine antigen. Analysis of the cellular immune responses of immunized, uninfected mice demonstrated that the vaccine induced a significant increase in CD4^+^ T cells producing IFN-γ, IL- 2, and tumor necrosis factor cytokines, indicating a Th 1-type immune response. Experimental infection of mice and hamsters demonstrated that, Leish-111f + MPL^®^-SE induced significant protection against *L. infantum* infection, with reductions in parasite loads of 99.6%, a level of protection greater than that reported for other vaccine candidates in animal models of visceral leishmaniasis (VL). The Leish-111f + MPL^®^-SE product reported in this study is the first defined vaccine for leishmaniasis in human clinical trials and has completed phases I and II safety and immunogenicity testing in normal, healthy human subjects[53]. A study by Skeiky *et al*.[22] reported that, MPL-SE formulated with rLeish-111f elicited protective immunity against *L. major* infection. This study concluded that, the demonstrated feasibility of manufacturing a single recombinant vaccine comprising multiple protective open reading frames and the potential use of MPL-SE as a substitute for IL-12, takes us closer to the realization of an affordable and safe *Leishmania* vaccine.

### CPG Oligodeoxynucleotide (CpG ODN)

Unmethylated CpG dinucleotide motifs present in bacterial genomes or synthetic oligodeoxynucleotides (ODNs) serve as strong immunostimulatory agents in mice[54] boosting the humoral and cellular response to coadministered antigens[55]. ODNs which contain immunostimulatory CG motifs (CpG ODN) can promote Th 1 responses, an adjuvant activity that is desirable for vaccination against leishmaniasis. BALB/c mice vaccinated with 20 µg of SLA plus 50 µg of CpG ODN showed a highly significant (P<5×10^−5^) reduction in swelling compared to SLA-vaccinated mice and enhanced survival compared to unvaccinated mice. The modulation of the response to SLA by CpG ODN was maintained even when mice were infected 6 months after vaccination .This study indicated that, CpG ODN was not an effective adjuvant for antibody production in response to SLA unless given together with alum, when it promoted the production of LgG2a, a Th 1-associated isotype. The study concluded that, with an appropriate antigen, CpG ODN would provide a stable, cost effective adjuvant for use in vaccination against leishmaniasis[56]. Similar to IL-12, the addition of ODNs (CpG) to the leishmanial antigens, TSA, LeIF and LmSTI-1, resulted in complete protection of susceptible mice against progressive disease, whereas no protection was observed in the absence of adjuvant[52]. Co-administering CpG ODN with heat-killed *Leishmania* vaccine, provided macaques with significantly increased protection against CL infection[55].

### Liposomes

Liposomes are microparticulate or colloidal carriers which form spontaneously when certain lipids are hydrated in aqueous media[11]. In a vaccine against murine CL, radiation-attenuated promastigotes were used for vaccination, mixed with dehydration-rehydration vesicle (DRV) liposomes as adjuvants. Results showed that, despite the development of significant T cell activation, this vaccination was not protective when the subcutaneous route was used. Vaccination through the intravenous route indicated that the antigen-liposome mixture produced resistance that was comparable with that of gamma-irradiated promastigotes in the BALB/c *L. major* model. The study concluded that the DRV liposomes, although a good adjuvant for inducing protective immunity to CL, are not able to overcome the requirement for an intravenous route of immunization with the leishmanial antigen preparation. Additionally, these data demonstrate a correlation between IL-3 and non-protection[57].

Leishmanial antigens extracted from the membranes of *L. donovani* promastigotes and injected intraperitoneally together with positively charged liposomes in hamsters and BALB/c mice, significantly protected these animals against infection with the virulent promastigotes[58]. These protected animals elicited profound DTH and increased levels of *Leishmania*-specific IgG antibodies. In another study, liposomes containing ALM and mixed with BCG were used as a vaccine against leishmaniasis in resistant C57 BL/6 mice. The DSV-ALM formulation was prepared by detergent solubilization (DSV) using egg lecithin and cholesterol (1:1 molar ratio) with BCG. The results indicated that skin test thickness in mice that received DSV-ALM-BCG was significantly higher than the other groups. Additionally, the result of cytokine assays showed that IFN-γ which is an indication of a Th 1 response was significantly higher in the group that received DSV-ALM-BCG than in the control groups. This study concluded that liposomes containing *Leishmania* antigens mixed with BCG could be used to induce a Th 1 response in resistant C57 BL/6 mice[11].

### Glucan

Glucan is a β 1,3 polyglucose derivative of baker's yeast. To study the protective efficacy of glucan, mice were immunized by a series of intravenous injections of formalin-killed *L. donovani* promastigotes alone and combined with glucan. Mice which received dead parasites and glucan exhibited resistance against challenge with viable *L. donovani* parasites. Animals which had been injected with glucan alone exhibited a lesser degree of resistance but injections of killed promastigotes alone conferred no measurable resistance against infection[59]. In a different study using CF1 mice, immunization via intravenous injections of dead promastigotes(5×10^6^) simultaneously with glucan (0.45 mg) elicited protective resistance, positive skin test responsiveness before and after challenge and increased antipromastigote antibody levels. In this study, injections of glucan alone induced a lesser degree of resistance against infection without significant skin test or humoral responsiveness[60].

A comparative study was performed on the protective efficacy of glucan as an adjuvant at a dose of 0.6 mg combined with killed promastigotes or soluble or particulate fractions (each at an equivalent dose of 1×10^7^ killed promastigotes) of the parasite in CF-1 mice. The results indicated that when these vaccine preparations were injected either intravenously or subcutaneously, glucan potentiated resistance against *L. donovani* infections as reflected by significant reductions in hepatic amastigote counts relative to infected control mice. The leishmanial antigens alone afforded no protection. Serum direct agglutination titres to leishmanial antigens were highest in all groups given the vaccine intravenously, whereas the DTH response to the antigen was positive only in groups immunized subcutaneously with glucan as an adjuvant. Some protection and immune response against visceral infection with the parasite was seen in groups vaccinated with glucan and soluble antigens. The protection afforded by glucan and particulate antigens of *L. donovani* more closely paralleled the resistance of mice treated with glucan and unfractionated killed promastigotes[61].

### Corynabacterium parvum (C. parvum)

Micro-organisms in bacterial infections and the administration of vaccines containing whole killed bacteria and some metabolic products and components of various micro-organisms have been known to elicit antibody responses and act as immunostimulants. The addition of such micro-organisms and substances into vaccines augments the immune response to other antigens in such vaccines. The commonly used micro-organisms, whole or their parts, are *Bordetella pertussis* components, cholera toxin, *Mycobacteria* and *Corynabacteria*[46].

*C. parvum* when used as an adjuvant in conjunction with a 46-kilodalton glycoprotein (M-2) of *L. amazonensis* appeared to be the most effective adjuvant in susceptible BALB/c and CBA and resistant C57BL/6 strains of mice when compared to both Freund incomplete and complete adjuvants and saponin. The level of protection varied with the mouse strain, although all animals received identical preparations of antigen and adjuvant. Immunization of CBA mice with the M-2 glycoprotein and *C. parvum* resulted in complete protection against a challenge infection of 10^4^ and 10^6^ late log-phase promastigotes of *L. amazonensis*. In the BALB/c strain, complete protection was observed in some of the immunized animals (28-50%). In the remaining mice the onset of infection was significant, while protective immunity for C57BL/6 was observed only at the low infection dose (10^4^ *L. amazonensis* organisms). The level of protection observed is reflected by the increased antibody response (Ig G1 and G2) developed to the M-2 molecule[62]. In a different study, intraperitoneal vaccination of C3h/He mice with *L. major* promastigote surface antigen-2 complex (PSA-2) with *C. parvum* as an adjuvant resulted in complete protection from lesion development after challenge infection with virulent *L. major*. In this study, significant protection was also obtained in the genetically susceptible BALB/cH-2k and BALB/c mice. This protection was as a result of a Th1 immune response as CD4^+^ T cells isolated from the spleens of vaccinated mice produced large amounts of IFN-γ but no detectable IL-4 upon stimulation with PSA-2 *in vitro*[63]. A different study demonstrated that, vaccination of mice three times intraperitoneally at 2 week intervals with 100 µg of recombinant parasite surface antigen 2 of *L. major* purified from *E. coli* (2.1-GST) and combined with *C. parvum* as an adjuvant did not induce protective immunity, despite the generation of strong Th1 responses[64]. A recent study utilizing *C. parvum* as an adjuvant indicated successful vaccination against *Leishmania chagasi* infection in BALB/c mice. In the study mice received two subcutaneous doses of *L. amazonensis* vaccine (100 µg) with *C. parvum* (250 µg) and a subsequent boost was done without adjuvant. One week later, mice were challenged with *L. chagasi* (1×10^7^). This vaccine caused a significant reduction in parasite load in liver and spleen and induced a high production of IFN-γ and IL-4 by spleen cells from vaccinated mice in response to *Leishmania* antigen[65].

### Saponins (Quil-A, ISCOM and QS-21)

Saponins are triterpene glycosides isolated from plants[43]. The most widely used saponin in adjuvant research is Quil-A and its derivatives, extracted from the bark of the *Quillaja saponaria* tree[66]. Quil-A is composed of a heterogeneous mixture of triterpene glycosides that vary in their adjuvant activity and toxicity. Saponins have been widely used as an adjuvant in veterinary vaccines. Partially purified fractions of Quil-A have also been used in immunostimulating complexes (ISCOM) composed of antigen, phospholipids, cholesterol and Quil-A fractions[7]. In a vaccine study using the dog, saponin used as an adjuvant when combined with *Leishmania braziliensis* promastigote protein elicited strong antigenicity related to the increases of anti-*Leishmania* IgG isotypes, together with higher levels of lymphocytes, particularly of circulating CD8^+^ T-lymphocytes, and *L. chagasi* antigen specific CD8^+^ T-lymphocytes[67]. Used in a concentration of 600 µg of *L. braziliensis* promastigote protein in 1 ml sterile saline, 1 mg of saponin was delivered subcutaneously. This candidate vaccine produced intense cell proliferation and increased nitric oxide during in vitro stimulation by *L. chagasi* soluble antigens[67].

In a study by Santos *et al*[68], the facos mannos ligand antigen of *L. donovani*, in combination with saponin, aluminium hydroxide (Al(OH)_3_) and Freund's incomplete adjuvant (FIA) was used in vaccines tested in an outbred murine model of visceral leishmaniasis, either through intraperitoneal or subcutaneous routes at dosages of 150 µg facos mannose ligand (FML) of *Leishmania* antigen, 100 µg saponin, 500 µg Al(OH)_3_ and 0.1 ml FIA. The humoral response was significantly higher in groups treated with FML + saponin or FML + Al(OH)_3_ than in controls both before and after the infection. A significant and specific reduction of parasitic load in relation to saline (85%, *P* < 0.01) and saponin (*P* < 0.025) controls, was seen in animals treated with FML + saponin by the intraperitoneal route. Coincidentally, with this reduction, an increase in antibodies of the IgG2a subtype was detected only in animals treated with FML + saponin intraperitoneally. Vaccination with FML + saponin was superior to other treatments and had no toxic effect due to saponin[68]. Mice vaccinated three times intraperitoneally at 2 week intervals with 0.5-1 µg of ISCOMS combined with 2.1-GST did not induce protective immunity despite the generation of strong Th1 responses[64]. ISCOMs have only been used in veterinary vaccines, partly due to their haemolytic activity and some local reactions[46].

### Freund's adjuvants

There are two Freund's adjuvants; incomplete and complete[46]. Freund's complete adjuvant (FCA) is a mixture of non-metabolizable oil (mineral oil), a surfactant (Arlacel A), and mycobacteria (*M. tuberculosis or M. butyricum*). It is prepared as a water-in-oil emulsion by combining one volume FCA with one volume aqueous antigen solution. In an emulsion, antigen is distributed over a large surface area thereby increasing the potential for interaction with relevant cells[69]. Antibody production is increased by FCA primarily because of the depot effect and nonspecific immunopotentiation of macrophages by surfactant and the mycobacteria. FCA combined with killed *Leishmania infantum* promastigotes was used as a *Leishmania* vaccine in dogs[70]. The volume of the vaccine was 100 µl (30 µl of promastigote preparation plus 70 µl of FCA) and was given subcutaneously in two different sites at the same time. Results indicated significantly increased phagocytosis, killing capacity and nitric oxide production by canine macrophages one month after vaccine administration with the increase also persisting up to 5 months later. In addition, the amount of IFN-γ in PBMC supernatants was significantly higher after vaccination, suggesting the proactive potential of this antigen-adjuvant combination[70]. Freund's adjuvants are considered to cause adverse reactions. The pathologic reaction to the Freund's adjuvants starts at the injection site with mild erythema and swelling followed by tissue necrosis, and intense inflammation[46].

Several other minor adjuvants have also been used in *Leishmania* vaccine studies. These include FIA, laurly-cystein and Salmonera typhimurium as reviewed by Rivier *et al* [71].

## CONCLUSION

It seems likely that adjuvants will be increasingly important as the science of *Leishmania* vaccine advances. First generation (killed) vaccines, which are relatively safe, have been made and they could be improved by the addition of appropriate adjuvants to provide longer lasting protection with fewer inoculations. Criteria involved in selecting the formulation for a given vaccine include the nature of the antigenic components, type of immune response desired, preferred route of delivery, avoidance of considerable adverse effects and stability of the vaccine. The optimally formulated adjuvant will be safe, stable before administration, readily biodegraded and eliminated, able to promote an antigen specific immune response, and inexpensive to produce. New vaccines are urgently needed for many infections caused by intracellular infections including HIV, leishmaniasis, malaria and tuberculosis. They will require a more sophisticated approach. These intracellular pathogens are well-adapted parasites with sophisticated mechanisms for evading immune responses. In developing vaccines for leishmaniasis, it will be necessary to consider adjuvants as sophisticated agents which can critically influence many parameters of immune responses including specificity, type, intensity, duration and genetic variability. The study of adjuvants is, in reality, the study of factors that control the expression of different types of immune responses. It would be exciting to discover an adjuvant that, when combined with a suitable antigen, will be able to induce an early, potent and long lasting *Leishmania*-specific cellular immune response.
